# Evaluation of adherence measures of antiretroviral prophylaxis in HIV exposed infants in the first 6 weeks of life

**DOI:** 10.1186/s12887-015-0340-9

**Published:** 2015-03-19

**Authors:** Alicia Catherine Desmond, Dhayendre Moodley, Catherine A Conolly, Sandra A Castel, Hoosen M Coovadia

**Affiliations:** Center for the AIDS Programme of Research in South Africa-CAPRISA, and Women’s Health and HIV Research Unit, Department of Obstetrics and Gynaecology, Nelson R Mandela School of Medicine, University of KwaZulu-Natal, Durban, South Africa; Biostatistics Unit, Medical Research Council, Durban, South Africa; Division of Clinical Pharmacology, Department of Medicine, University of Cape Town, Cape Town, South Africa; Maternal Adolescent and Child Health (MatCH), University of the Witwatersrand, Johannesburg, South Africa

**Keywords:** Adherence measures, Infants, Antiretroviral prophylaxis, Maternal verbal report, Pharmacy returns

## Abstract

**Background:**

Adherence to an antiretroviral regimen is imperative for treatment success in both HIV infected adults and children. Likewise, adherence to antiretroviral prophylaxis is critical in HIV prevention. Studies on pediatric adherence are limited, particularly the prophylactic use of antiretroviral drugs and treatment adherence in very young infants. The HIV Prevention Trials Network (HPTN) 046 study (Clinical Trial Registration NCT00074412) determined the safety and efficacy of an extended regimen of nevirapine suspension in infants born to HIV-1 infected women for the prevention of vertical HIV transmission during breastfeeding. As per protocol, adherence to nevirapine prophylaxis was measured by maternal verbal reports. In addition, the pharmacy assessed the unused returned suspension. The aim of this sub-study was to determine the reliability of maternal verbal reports in measuring adherence to antiretroviral prophylaxis in infants in the first 6 weeks of life and evaluating the unused returned nevirapine as an alternative method of measuring adherence.

**Methods:**

Maternal verbal reports and pharmacy returns indicative of “missed < 2 doses” were evaluated against a plasma nevirapine concentration of >100 ng/ml in a subgroup of infants at 2, 5 and 6 weeks of age. Plasma nevirapine concentration of >100 ng/ml was used as a marker of adherence (10 times the in vitro IC_50_ against HIV).

**Results:**

Adherence was 87.7% (maternal verbal report) and 71.3% (unused returned medication), as compared to 85.6% by plasma nevirapine concentration. Evaluated against plasma nevirapine concentration <100 ng/ml, the sensitivity and specificity of maternal verbal reports to detect a missed dose in the last 3 days were 75% and 78% (p = 0.03) respectively. Overall, among infants who were classified as adherent based on missed doses by maternal verbal reports and unused returned medication, 88.4% and 87.4% of infants attained a nevirapine concentration above 100 ng/ml respectively.

**Conclusion:**

Maternal verbal reports are a reliable measure of adherence to infant antiretroviral prophylaxis in the first 6 weeks of life and could be useful in assessing adherence to antiretroviral treatment in infants younger than 6 weeks. In the absence of resources or expertise to determine plasma drug concentration, we would recommend random assessments of unused returned medication.

## Background

An estimated 2.3 million people were newly infected with HIV globally in 2012, of which 260 000 were children [1]. The infection was averted in more than 670 000 children from 2009 to 2012 due to the accessibility of services to prevent mother-to-child transmission [2] which includes the provision of antiretroviral (ARV) drugs that are taken by the mother during pregnancy and delivery and her newborn infant [3].

The number of women and infants that have been receiving ARV drugs for this purpose has been steadily increasing [4]. An estimated 88% of HIV positive pregnant women and 56% of HIV exposed infants received ARV prophylaxis in 2009 alone in South Africa [5]. Early studies have demonstrated that infant ARV prophylaxis in the first 6 weeks of life could significantly reduce risk of intrapartum or early breastfeeding transmission of HIV [6]. Consequently, evidence based prevention of mother-to-child transmission (PMTCT) guidelines currently recommend that nevirapine suspension must be given to all HIV-exposed infants at birth and for 6 weeks thereafter as post-exposure prophylaxis for intrapartum and early breastfeeding transmission, irrespective of feeding practice or maternal ARV treatment options [7-9].

Adherence to a PMTCT regimen undoubtedly contributes to its efficacy and hence adherence would be imperative to ensure that the target of eliminating new pediatric HIV infections by 2015 is met [10,11]. Adherence is defined as the extent to which prescribed medication is taken by patients and is measured by direct and indirect methods [12]. Direct methods include biological assays of an active drug in the blood or body fluids and directly observed therapy (DOT). Indirect measures include pill counts, Medication Event Monitoring System (MEMS), pharmacy refills and verbal reports by caregivers, patients and physicians [13-16]. Each method has its advantages and disadvantages [17].

Adherence and various measures of adherence are well documented for the adult population in both low to middle income and high income countries [18-21]. However studies on pediatric adherence are limited [14] particularly the prophylactic use of ARV drugs.

The HPTN046 prospective cohort study (Clinical Trial Registration NCT00074412) was conducted from June 2008 to March 2010 to determine the safety and efficacy of an extended regimen of nevirapine in infants born to HIV-1 infected women for the prevention of vertical HIV transmission during breastfeeding [8]. In this clinical trial, adherence to nevirapine prophylaxis in infants was assessed by maternal verbal reports. In this sub-study we evaluated two indirect measures (maternal verbal reports and unused returned nevirapine medication) against a direct measure (plasma nevirapine concentration) of adherence in these HIV exposed infants receiving daily nevirapine prophylaxis for the first 6 weeks of life. The overall aim of this evaluation was to ascertain the reliability of maternal verbal reports and weight measurements of unused returned nevirapine suspension as an alternative method of measuring adherence.

## Methods

### Study design, setting and population

This was a retrospective cohort study. Data was retrieved from the HPTN046 study [8]. This study was conducted at the Umlazi Clinical Research site located on the grounds of the Prince Mshiyeni Memorial Hospital (PMMH) in Umlazi Township. HIV exposed breastfed infants enrolled in the HPTN046 study received nevirapine suspension (10 mg/ml) for the first 6 weeks of life, and at 6 weeks eligible infants who remained HIV negative were randomized to receive either an extended regimen of nevirapine or placebo until 6 months of age or until cessation of breastfeeding, whichever was earliest. Study visits in the first 6 weeks of life after enrolment (day 3-7 after birth) were scheduled for 2, 5 and 6 weeks. The dose at enrolment began at 0. 6 ml (6 mg) daily until the 2 week visit at which point the dose increased to 1. 5 ml (15 mg) given as a daily dose until the 5 week visit. At this visit, the dose was then increased to 1. 8 ml (18 mg) daily until day 42 (birth = day 0). Participants who were on study drug “hold” for safety evaluations were excluded from the analysis.

The HPTN046 study was approved by the University of Kwazulu-Natal Ethics Committee (T190/03) and the Medicines Control Council. Mothers provided written informed consent at entry into the HPTN046 study. At each visit in the main study, participants were clinically examined, blood specimens drawn for laboratory investigations and storage for further research. This sub-study was a retrospective cohort data analysis in which data was obtained from the HPTN 046 study. It was approved by the University of Kwazulu-Natal Ethics Committee and the HPTN046 study team.

### Measurement of adherence by maternal verbal reports

Information regarding infant adherence was obtained from mothers using a structured questionnaire. Questions included whether infants missed doses since the previous visit, the number of days missed and the reason for missed doses. Other information obtained included maternal socio-demographic characteristics. The relevant data for this sub-study were extracted from the main electronic database at three different time points, at the 2 week, 5 week and 6 week visit for this study. Participants who reported missing two or more doses were classified as non-adherent. The association between demographic characteristics and missed doses reported by mothers was determined for the longest period (2-5 week visit). Mothers provided the dates of the last 3 doses given to the infant and they also reported on whether the infants missed 2 or more days in a row in the period between visits. Responses to both questions were utilized in the assessment of adherence.

### Measurement of adherence by assessment of unused returned Nevirapine

Nevirapine suspension was dispensed to mothers at each visit. Women received instructions for administration of the suspension to their infants. These instructions were also printed on the labels attached to the bottles of medication. Adherence counseling was performed after each dispensing. The number of bottles dispensed varied at each visit according to the HPTN046 protocol. Used bottles containing remaining nevirapine suspension were returned by participants at the next visit. Each bottle was weighed independently by pharmacists. Six full bottles were weighed and an average was calculated to obtain an average weight of 29.4 g for a full bottle of nevirapine suspension. The number of missed doses was calculated by dividing the difference between the volume used and volume that should have been used by the daily dosage. This figure was thereafter adjusted for potential spillage and adhesion of suspension to the syringe walls and tip by adding one dose to the measured weight of the suspension in the bottle.

### Plasma Nevirapine concentration

Nevirapine concentrations were determined in stored plasma samples of adequate volumes in a subgroup of participants at the 5 and 6 week visits for the purpose of comparing maternal verbal reports to weighed returned medication. Concentrations were determined by LC-MS/MS (Division of Clinical Pharmacology, University of Cape Town). The assay was validated according to FDA and EMA guidelines. Plasma samples were extracted and chromatographic separation was achieved on a Luna 5 μm PFP (2), 100 A, 50 mm × 2 mm analytical column. An AB Sciex API 4000 mass spectrometer was operated at unit resolution in the multiple reaction monitoring (MRM) mode, monitoring the transition of the protonated molecular ions at m/z 266.9 to the product ions at m/z 198.2 for Nevirapine, and monitoring the transition of the protonated molecular ions at m/z 270.1 to the product ions m/z 229.1 for the stable isotope labeled nevirapine internal standard. The calibration curve fitted a quadratic (weighted by 1/concentration^2^) regression over the ranges 0.0195 – 20.0 μg/ml. Nevirapine concentration above 100 ng/ml was used as a marker for adherence (10 times the in vitro IC_50_ against HIV) [22,23].

### Statistical analysis

Categorical variables were summarized as percentages. Frequency distributions of continuous variables did not meet the Shapiro-Wilk W test for normal data therefore medians and inter-quartile ranges (IQR) were used as summary measures. These variables were also dichotomized using commonly accepted cut-points. Subgroups were compared using Chi Square tests or Fisher’s exact test for categorical variables and Odds Ratio and 95% confidence interval reported. Independent associations with missed dose reporting were examined using a stepwise logistic regression model which includes all variables. Two sided *P* < 0.05 was considered statistically significant. All analyses were performed using EPI-info (version 3.4.3) and Stata (version 12).

## Results

### Study population characteristics

A total number of 225 mother-infants pairs were included in this sub-study analysis. Maternal ages ranged from 18 years to 42 years with a median age of 25.7 years (IQR 22.5-29.7) The majority of women (90.7%) were single and not living with a partner and first pregnancies were reported in one in four women. Literacy levels amongst the women were relatively high with 93.3% achieving grade 7. Almost half of the women (45.5%) were in an advanced stage of HIV (CD_4_ ≤ 350 cells/mm^3^) and women receiving triple ARV’s as treatment had a CD4 count of less than 200 cells/mm^3^ [24]. Two thirds of the women (64.9%) had normal vaginal deliveries and 79 (35.1%) had caesarean sections. The mean birth weight was 3.1 (range 2-4.3) and 11.6% of infants (26/225) weighed under 2.5 kg.

### Maternal verbal reports

**Figure 1:**
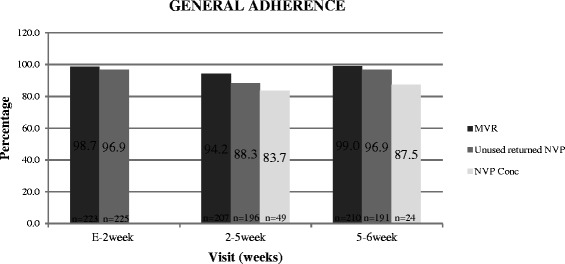
**Adherence based on missing ≥ 2 doses: Maternal verbal report (MVR), Unused Returned Nevirapine (NVP) and > 100 ng/ml plasma NVP concentration.**

Adherence was assessed at the 2 week (n = 223), 5 week (n = 207) and 6 week visits (n = 210). Three (1.3%), twelve (5.8%) and two (1.0%) women reported that 2 or more doses were missed at the 2, 5 and 6 week visits respectively. Adherence was calculated as 98.7% (220/223), 94.2% (195/207) and 99.0% (208/210) at 2, 5 and 6 week visits respectively (Figure 1). Reasons for missed doses amongst the 17 women that reported 2 or more missed doses included difficulties in drawing medication from the bottle (11.8%), misunderstanding (mothers stopped dosing the infant as they were not aware that they could re-use syringes provided) (11.8%), mother was ill or hospitalized (17.6%), mother forgot (5.9%), missed visit (5.9%), mother stopped breastfeeding (29.4%), mother thought that the study drug was expired (5.9%), disclosure issue (5.9%) and lack of support (5.9%). Adherence in the subgroup that had plasma nevirapine concentrations determined at the 5 week (n = 49) and 6 week visits (n = 24) was 79.6% (39/49) and 95.8% (23/24) respectively. Average adherence for this subgroup was 87.7% with maternal verbal report (5 and 6 week visits).

### Maternal and infant characteristics associated with maternal verbal report missed doses at the 5 week visit

In general, younger women who were single and were primigravida more often reported a missed dose than older women with a partner and who were multiparous however these differences were not statistically significant. More women that were 25 years and younger reported a missed dose, 18% (21/116) versus 15% (15/102) for women above 25 years of age (OR 1.3; 95% CI 0.6-3.0). Single women were also more likely to report missed doses, 18% versus 5% in married/living with a partner (OR 4.1; 95% CI 0.5 – 31) and (OR 1.3; 95% CI 0.5 – 2.9). More women pregnant for the first time reported a missed dose 19% (9/48) compared to that of a multiparous woman, 16% (24/154) OR: 1.3 (95% CI 0.5-2.9). Women receiving an ARV regimen either received medication for PMTCT (1), a triple ARV regimen (2) or medication for PMTCT and a triple ARV regimen (3). There were also mothers that received no ARV medication (0) (Table 1). Nevirapine concentrations were determined in 8 infants whose mothers were on a triple ARV regimen containing nevirapine. The average concentration amongst these women was 1709 ng/ml. The average concentration amongst those infants whose mothers were not exposed to nevirapine was 1702 ng/ml hence exposure to maternal nevirapine did not alter the plasma concentrations in the infants. After controlling for possible confounding variables such as age, marital status, education, HIV clinical stage, ARV regimen and infant birth weight, a multivariate logistic regression showed no variables independently related to reporting of missed doses.

**Table 1:** **Maternal and infant characteristics in association with maternal verbal reports (MVR) missed dose at the 5 week visit**

	**MVR MISSED DOSE**				
	**Yes (n = 36)**	**No (n = 182)**			
	**n (%)**	**n (%)**	**OR**	**95% CI**	**P value**
**Age**					
≤25 years	21 (18)	95 (82)	1.3	(0.6 - 3)	
>25 years	15 (15)	87 (85)	ref		0.6
**Marital status (n, %)**					
Single	35 (18)	163 (82)	4.1	(0.5 – 31)	0.2
Married	1 (5)	19 (95)	ref		
**Education**					
≤ Grade 7	1 (7)	13 (93)	ref		
> Grade 7	35 (17)	169 (83)	2.7	(0.3 – 21.3)	0.3
**Parity (n, %)**					
Primigravida	9 (19)	39 (81)	1.3	(0.5 – 2.9)	0.6
Multiparous	24 (16)	130 (84)	ref		
**HIV clinical stage and ARV**					
CD_4_ ≤ 350 (n%)	17 (17)	81 (83)	1.1	(0.5-2.3)	0.8
CD_4_ > 350 (n%)	19 (16)	101 (84)	ref		
**WHO Clinical stage (n, %)**					
1	33 (16)	170 (84)	1.2	(0.1 - 10)	0.9
2 or 3	1 (14)	6 (86)	ref		
**Receiving ARV regimen (n,%)**					
0, 1	29 (18)	136 (82)	1.4	(0.6 - 3.4)	0.5
2, 3	7 (13)	46 (87)	ref		
**Mode of delivery (n, %)**					
Normal	25 (17)	118 (83)	1.2	(0.6 – 3.0)	0.6
C/S	11 (15)	64 (85)	ref		
**Birth weight (n, %)**					
2.0 - 2.5 kg	4 (15)	22 (75)	ref		
>2.5 kg	32 (17)	160 (83)	1.1	1.1 (0.4 - 3)	0.9

### Adherence based on unused returned Nevirapine

The number of missed doses was calculated at the 2 week (n = 225), 5 week (n = 196) and 6 week (n = 191) visits. Seven (3.1%), twenty three (11.7%) and six (3.1%) infants missed 2 or more than doses at the 2, 5 and 6 week visits respectively. Adherence based on returned nevirapine at the 2, 5 and 6 week visits was estimated as 96.9% (218/225), 88.3% (173/196) and 96.9% (185/191) respectively (Figure 1). Adherence in the subgroup that had plasma nevirapine concentrations determined at the 5 week (n = 49) and 6 week (n = 24) visits was 59.2% (29/49) and 83.3% (20/24) respectively. Average adherence for unused returned nevirapine was 71.3% for this subgroup (5 and 6 week visits).

### Plasma nevirapine concentration

The median nevirapine concentrations were 1620 ng/ml (IQR 1000-2220 ng/ml) and 1380 ng/ml (IQR 448-2835 ng/ml) at the 5 week (n = 49) and 6 week (n = 24) visits respectively. 83.7% (41/49) (95%CI 70.3-92.7) and 87.5% (21/24) (95% CI 67.6-97.3) of the infants had a plasma nevirapine concentration of more than 100 ng/ml at 5 wk and 6 wk respectively (Figure 1). Average adherence determined by nevirapine concentration was 85.6% (5 and 6 week visits).

### Agreement between plasma nevirapine concentration, maternal verbal reports and unused returned nevirapine in identifying missed doses

The sensitivity of maternal verbal report in the last 3 days and unused returned nevirapine to detect missed doses was exactly the same at the 5 week visit (75% [95% CI 35-97]) p = 0.9. The specificity was 78% [95% CI 62-89] at the 5 week visit for maternal verbal report and was significantly higher than nevirapine returns at this visit (42% [95% CI 26-58) (p =0.002) (Table 2). The sensitivity for unused returned nevirapine to detect missed doses at the 6 week visit was 100% however there were only 3 participants in which the concentration was less than 100 ng/ml. Again, the specificity was higher (70% [95% CI 46-88]) for the maternal verbal report than the unused returned nevirapine (38% [95% CI 18-62]) at the 6 week visit.

**Table 2:** **Agreement between nevirapine concentration and maternal verbal reports (MVR) and unused returned nevirapine (NVP) in identifying missed doses at the 5 week visit**

**Adherence measure**	**Sensitivity**	**Specificity**	**PPV**	**NPV**	P-value *
MVR n (%) 95% CI	6/8 (75%) (35; 97)	32/41 (78%) (62; 89)	6/15 (40%) (16; 68)	32/34 (94%) (80; 99)	0,03
unused returned NVP n (%) 95% CI	6/8 (75%) (35; 97)	17/41 (42%) (26; 58)	6/30 (20%) (8; 39)	17/19 (89%) (67; 99)	<0.001

*P-value represents the comparison between maternal verbal reports/unused returned nevirapine and NVP concentration.

### Relationship between maternal verbal reports, unused returned nevirapine and nevirapine concentration in adherent and non-adherent patients

At the 5 week visit, 89.7% of infants whose mothers reported adherence (missing one dose or not missing any doses) had a plasma nevirapine concentration of greater than 100 ng/ml. The same percentage (89.7%) of infants that were categorized adherent as calculated from the unused returned nevirapine had a concentration of greater than 100 ng/ml. At the 6 week visit, 87% of infants whose mothers reported adherence had a plasma nevirapine concentration of greater than 100 ng/ml and 85% as calculated on the unused returned nevirapine (Table 3).

**Table 3:** **Relationship between maternal verbal report (MVR), unused returned nevirapine (NVP) and NVP concentration in adherent patients (missed < 2 doses)**

**Visit**	**Infants classified as adherent by MVR (n)**	**Infants classified as adherent by MVR and have > [100 ng/ml] n (%)**	**Infants classified as adherent by MVR and have < [100 ng/ml] n (%)**
5 week	39	35 (89.7%)	4 (10.3%)
6 week	23	20 (87.0%)	3 (13.0%)
	**Infants classified as adherent by unused NVP (n)**	**Infants classified as adherent by unused NVP and have > [100 ng/ml] n (%)**	**Infants classified as adherent by unused NVP and have < [100 ng/ml] n (%)**
5 week	29	26 (89.7%)	3 (10.3%)
6 week	20	17 (85.0%)	3 (15.0%)

For those infants whose mothers reported missing 2 or more doses at the 5 week visit, 60% had a plasma nevirapine concentration of greater than 100 ng/ml. 75% of those that missed 2 or more doses according to the unused returned nevirapine had a plasma nevirapine concentration of greater than 100 ng/ml. At the 6 week visit, all infants that missed 2 or more doses as per maternal verbal report and unused returned nevirapine had a plasma concentration of greater than 100 ng/ml.

### Correlation between maternal verbal reports and unused returned nevirapine at the longest visit

A comparison of the percentages during the longest period (2-5 week) reveals that there is no significant difference in the doses missed for nevirapine returns (21.3%) (44/207) and maternal verbal reports (15.5%) (32/207) (p = 0.1). This indicates good agreement between the two measures (Mcnemars chi2 (1) = 2.67, Prob > chi2 = 0.1025).

## Discussion

In this study, adherence to antiretroviral prophylaxis in HIV exposed infants in the first 6 weeks of life was assessed using two indirect methods (verbal reports and unused returned medication) in association with a direct method (plasma concentration of medication). Prophylactic adherence determined by maternal verbal reports exceeded 90% at all clinic visits in the 6 week period. Adherence as measured by unused returned medication was marginally lower at short visit intervals but significantly lower when medication was returned after a long visit interval.

Verbal reports are often relied upon to assess adherence to medication [25] among adults and children and this measure of adherence has been previously evaluated in treatment studies. There are no known evaluation studies of this measure for use in assessing adherence to a prophylactic regimen in children. Previous studies have shown that adherence reported by caregivers is an inflated figure in comparison with other measures in HIV infected children [26]. This has also found to be the case in the adult population with self-reported adherence [27]. In our study, adherence as per maternal verbal reports was more comparable to adherence determined by the direct method of plasma nevirapine concentration in the subgroup of participants than unused returned nevirapine. The subgroup was the small number of participants for who adequate volumes of plasma was available to determine nevirapine concentrations. A difference can be noted in adherence based on maternal verbal report in the nevirapine level subgroup (79.6%) in comparison to the overall group (94.2%) at the 5 week visit. This is because the subgroup included almost all (10) of the patients that were non-adherent in the general group (12). Agreement between plasma nevirapine concentration and maternal verbal reports was found to be slightly better than the agreement between plasma nevirapine concentration and unused returned nevirapine at the visit after the longest gap. The specificity for the unused returned nevirapine was much lower than the maternal verbal reports due to the fact that the unused returned nevirapine was confounded by the longer period. This was one of the limitations of the study. It was not possible to investigate if the last few doses were missed when calculating adherence using weights of unused returned nevirapine, whereas information that mothers provided included the last 3 doses that the infant had taken and this could therefore be used in determining agreement between plasma nevirapine concentration and maternal verbal reports. This is the case because plasma drug concentration is a reflection of the last few doses that a patient has taken [28]. Factors that affect plasma drug concentration levels [26] include inter-patient variation and the timing of drawing a blood sample in relation to the last dose taken [28]. It has previously been shown that the decay in plasma concentrations of nevirapine within dosage intervals is fairly small therefore randomly taken samples are adequate [29,30]. Due to the fact that nevirapine has a long elimination half-life, great intra-individual variations in plasma concentration was not expected [29,30]. Nevirapine is metabolised primarily by cytochrome P450 CYP3A4 and CYP2B6 [31]. Adult studies have reported that the CYP 2B6 516G → T polymorphism is associated with elevated nevirapine plasma concentrations in HIV infected patients [32-34]. Similarly, studies conducted in infants have reported that those infants with the CYP2B6 516 TT (homozygous mutants) genotype had a decreased oral clearance of nevirapine in comparison to those with 516GT (heterozygous) and 516 GG (wildtype) genotypes [35,36]. It was also previously reported that the CYP2D6 enzymes play a role in metabolism in HIV infected children [37]. It is possible that these polymorphisms influence nevirapine concentrations in the infants in this study, however this was not assessed.

It is not clear from previous research exactly how many doses can be missed consecutively in the pediatric population for the concentration to fall below the required level (100 ng/ml -10 times the in vitro 50% concentration against HIV-1). It was previously found in a study where trough nevirapine levels were determined, that in order to maintain a therapeutic target of 100 ng/ml in 100% of participants, infants had to receive a once daily dose. However a twice weekly dose given on the first and fourth days of each week also maintained a therapeutic target of 100 ng/ml in 62 of 65 samples (95.4%) [38]. It can be deduced from this study and our sub-study that infants who miss two to three consecutive doses can still obtain the nevirapine concentration of 100 ng/ml, therefore they would need to receive at least twice weekly doses given a maximum of 72 hours apart.

In our study, the maternal verbal report had given us a better indication of adherence in comparison to the measure of unused returned nevirapine. It was entirely the caregiver’s responsibility to ensure good adherence in this study as infants assessed were between the ages of 3 days and 6 weeks of age. Mothers (caregivers) were required to report adherence. Other studies that assessed adherence by caregiver reports utilized 3 day recall [13,26,39-41] 4 day recall [25] and 24 hour recall methods [16]. In comparison, our study required mothers to also report on doses missed since the last visit which could have been 3 to 26 days earlier. Caregiver reports have been found to be a reliable measure in the assessment of adherence in some studies [16,42].

The direct method of determining plasma nevirapine concentrations has been used previously to detect non adherence [43]. In our study, it was used as a gold standard, for the purpose of assessing other measures of adherence. However the limitation of this study is that plasma nevirapine concentrations were determined in a subgroup of participants.

Caregiver reports are utilized to a greater extent than other adherence measures in routine practice. It has previously been reported that using multiple measures of adherence is more beneficial than using a single measure [44].

## Conclusion

We have concluded that maternal verbal reports are a reliable measure of adherence to infant antiretroviral prophylaxis in the first 6 weeks of life and could be useful in assessing adherence to antiretroviral treatment in infants younger than 6 weeks. In the absence of resources or expertise to determine plasma drug concentration, we would recommend random assessments of unused returned medication.
